# The Non-linear Trajectory of Change in Play Profiles of Three Children in Psychodynamic Play Therapy

**DOI:** 10.3389/fpsyg.2016.01494

**Published:** 2016-10-10

**Authors:** Sibel Halfon, Alev Çavdar, Franco Orsucci, Gunter K. Schiepek, Silvia Andreassi, Alessandro Giuliani, Giulio de Felice

**Affiliations:** ^1^Department of Psychology, Istanbul Bilgi UniversityIstanbul, Turkey; ^2^Division of Psychoanalysis, University College LondonLondon, UK; ^3^Research Institute of Synergetics and Psychotherapy, Paracelsus Private Medical University of SalzburgSalzburg, Austria; ^4^Clinical and Psychodynamic Psychology Department, Sapienza University of RomeRome, Italy; ^5^Environment and Health Department, National Institute of HealthRome, Italy; ^6^Institute for Complexity Studies, Sapienza University of RomeRome, Italy

**Keywords:** psychodynamic play therapy, play assessment, play profiles, complexity science, non-linear dynamics

## Abstract

**Aim:** Even though there is substantial evidence that play based therapies produce significant change, the specific play processes in treatment remain unexamined. For that purpose, processes of change in long-term psychodynamic play therapy are assessed through a repeated systematic assessment of three children’s “play profiles,” which reflect patterns of organization among play variables that contribute to play activity in therapy, indicative of the children’s coping strategies, and an expression of their internal world. The main aims of the study are to investigate the kinds of play profiles expressed in treatment, and to test whether there is emergence of new and more adaptive play profiles using dynamic systems theory as a methodological framework.

**Methods and Procedures:** Each session from the long-term psychodynamic treatment (mean number of sessions = 55) of three 6-year-old good outcome cases presenting with Separation Anxiety were recorded, transcribed and coded using items from the Children’s Play Therapy Instrument (CPTI), created to assess the play activity of children in psychotherapy, generating discrete and measurable units of play activity arranged along a continuum of four play profiles: “Adaptive,” “Inhibited,” “Impulsive,” and “Disorganized.” The play profiles were clustered through *K*-means Algorithm, generating seven discrete states characterizing the course of treatment and the transitions between these states were analyzed by Markov Transition Matrix, Recurrence Quantification Analysis (RQA) and odds ratios comparing the first and second halves of psychotherapy.

**Results:** The Markov Transitions between the states scaled almost perfectly and also showed the ergodicity of the system, meaning that the child can reach any state or shift to another one in play. The RQA and odds ratios showed two trends of change, first concerning the decrease in the use of “less adaptive” strategies, second regarding the reduction of play interruptions.

**Conclusion:** The results support that these children express different psychic states in play, which can be captured through the lens of play profiles, and begin to modify less dysfunctional profiles over the course of treatment. The methodology employed showed the productivity of treating psychodynamic play therapy as a complex system, taking advantage of non-linear methods to study psychotherapeutic play activity.

## Introduction

The aim of this article is to demonstrate how a comprehensive method of play assessment can be applied to long-term psychodynamic play therapy sessions and to illustrate how this method can enhance the understanding of psychotherapy process. The process of change in psychotherapy is assessed through a repeated systematic assessment of the “play profiles” of three children who share similar demographic, diagnostic characteristics, and treatment courses. Play profiles summarize each child’s pattern of play in each session using items from the Children’s Play Therapy Instrument (CPTI; Kernberg et al., 1998) created to assess the play activity of children in psychotherapy. Each profile reflects coping and adaptive strategies used by children and reveals an understanding of their social and internal world. The profiles include discrete and measurable units of play activity arranged along a continuum of four clusters: “Adaptive,” “Inhibited,” “Impulsive,” and “Disorganized.” In line with the principles of therapeutic change in psychodynamic therapy, the evolution of the play profiles in treatment are studied through a complex system design which analyzes the non-linear and non-stationary trajectory of clinical improvement across time. The main hypothesis is to test whether there is a decrease in the “dysfunctional” play profiles around the middle of the treatment followed by a more functional reorganization in the last phase of psychotherapy. A further question concerns how this reorganization takes place and the indexes (if any) predicting it.

Shirk and Burwell (2010), in a recent review of core change mechanisms in psychodynamic process research, indicated that the changes in the child’s capacity to play represent important targets for investigation. In this view, playing itself is a main agent of change (Winnicott, 1971; Target et al., 2005) which is also supported by a large body of developmental research indicating that play is critical for the child’s cognitive, affective and social development (see Russ, 2004; for a review). Child’s behaviors in play signify meaning and serve a communicative function reflective of the child’s problems, conflicts, coping mechanisms, relationships and representations (Fonagy and Target, 2003). The aim of psychotherapy is to help the child build a rich and coherent play narrative, imagine the inner lives of the play characters and find solutions in play that can contain the intense feelings generated and cope with problematic situations (Slade, 1994). As the child learns to embrace the “pretend mode” (Fonagy and Target, 1998), the space between reality and fantasy that allows for transformations, he can flexibly use different patterns of play with different coping strategies to work on his issues in psychotherapy.

Even though there is substantial evidence that play based therapies produce significant change across a variety of childhood emotional and behavioral problems, most of these studies only report successful outcome without elucidating the specific links between play processes and treatment effectiveness (see Bratton et al., 2005; for the most recent meta-analysis of play therapy). In order to understand the specific pathways associated with change, one needs to focus on the processes that take place in psychodynamic child psychotherapy, so that what goes on in treatment itself can be related to changes at outcome (Shirk and Burwell, 2010).

**FIGURE 1:**
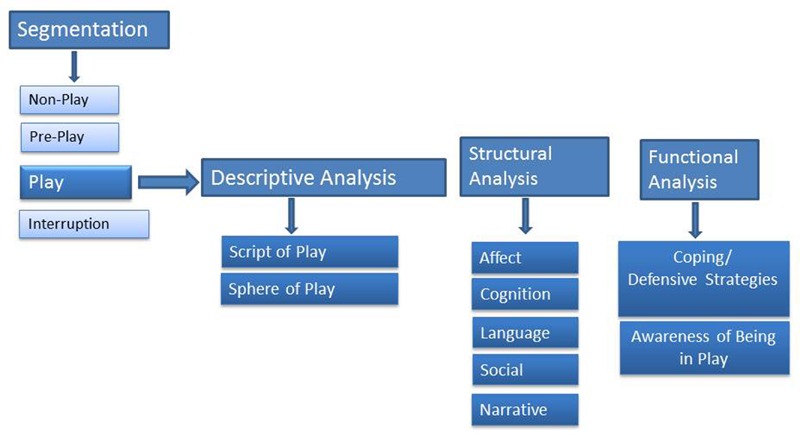
**Dimensions of Children’s Play Therapy Instrument (CPTI)**.

In more recent years, there has been some effort to categorize the quality of children’s play in psychotherapy and though sparse, there are a number of play measures with various degrees of empirical support (see Russ and Niec, 2011; for a review). Amongst these measures, CPTI (Kernberg et al., 1998) is a psychodynamically informed tool that can comprehensively assess the play activity of children with a clinical diagnosis. The scale involves three steps (see **Figure 1**): In the first step, the child’s activity in the session is rated indicating the presence of one, or more, of the following observations: Pre-play; Play activity; Non-play; Interruption. Going forward *only play activity* is rated through a Descriptive Analysis of play activity taking into account the category of the play activity (e.g., gross motor, fantasy, game play, etc.), the child’s capacity to initiate and facilitate play, (i.e., the child’s autonomy in play), and the sphere of play (where the play takes place). The instrument also assesses the structure of the play in terms of affective components (types of affect expressed in play and affect regulation strategies), cognitive components (how objects and people are represented in play), and the themes of play and the child’s use of language. In the Functional Analysis, the instrument assesses coping and defensive strategies, as well as a rating of the degree of the child’s subjective awareness of himself/herself as a player.

An important focus of CPTI has been to examine changes in a child’s activity in play sessions that occur over time (Chazan, 2000, 2001, 2002; Chazan and Wolf, 2002; Chari et al., 2013). The descriptive, cognitive, affective, and functional components shed light on different aspects of play on which a child can show improvement over the course of psychotherapy. Segmentation provides information regarding the overall progression of child play activity within the therapeutic hour and can be used to assess how much the child can engage in the play activity over the course of treatment. Chazan (2000, 2001, 2002) found that children who showed significant symptomatic improvement over the course of therapy also showed gains in the percentage of time spent in play activity as well as less time spent in non-play. The descriptive components help assess the child’s capacity to initiate the play, which is associated with the child’s sense of agency and freedom to play out his internal world in the presence of an adult using different mediums. In clinical populations where distressful or aggressive themes are more dominant, inhibition and disruptions are more clearly observed in carrying out of play to its completion (Howe and Silvern, 1981; Russ and Niec, 2011). Chazan (2000, 2002), Chazan and Wolf (2002), Chari et al. (2013) found that compared to pre-treatment, children at the end of treatment show more initiative in beginning and facilitating play and less inhibition of the play activity.

The affective components are a measure of the types and range of emotions exhibited by the child in his/her play, which are also reflective of feelings in his/her life. Perry and Landreth (1991), Chazan (2000, 2001, 2002) and Chari et al. (2013) found that over the course of treatment children show gains in pleasurable affect, less emotional discomfort as well as greater capacity for smooth transitions and regulation of affect. The cognitive components assess the structure of the representational world, that is the multiple representation of oneself in interaction with others (Sandler and Rosenblatt, 1962). This indicates the degree to which a child is capable of creating narrative structures to represent affect-laden relationships. Children with clinical problems are capable of simpler representations, where they may bring a solitary role to the play world, remaining more centered upon themselves in an egocentric fashion. Alternatively, the therapist or toys may be animated only as recipients, or extensions of the child’s activities (Slade, 1994; Chazan, 2002). As the child uses the therapeutic play field to bring problematic scenarios and work on them with the help of the therapist, more complex representations emerge, with several characters in interacting roles, taking into account different generational and familial dynamics. In doing this, the child creates narrative structures that represent different relationships. In effect, Chazan (2000, 2002) and Chari et al. (2013) have also documented significant improvement in cognitive dimensions over the course of treatment. The concept of coping-defensive components takes on concepts from Vaillant et al. (1986), Perry (1990), and Kernberg (1994) and assess the spectrum of coping strategies and defenses that characterize the child’s functioning in play. The prominence of adaptive defenses is characteristic of healthy coping and children are found to show more adaptive play strategies over the course of treatment (Chazan, 2002; Chari et al., 2013).

Apart from these individual indices, with the use of the CPTI, it is possible to identify patterns of organization among these CPTI play variables that contribute to play activity, resulting in *profiles* of play (Chazan, 2002). These profiles reflect a specific child’s experience of himself/herself while playing as an outward expression of his inner thoughts and feelings, as well as his strategies for coping and adaptation. They can be understood as the child’s trial efforts to resolve conflict, overcome obstacles and figure out social situations (Chazan, 2002). Chazan (2009) has identified four groups of children who share the same distribution of subscale variables corresponding to the following play profiles: Adaptive, Impulsive, Inhibited, and Disorganized. These could be reliably differentiated from others and also showed support for construct validity (Chazan, 2012). Each profile condenses *different coping strategies* used by the child to deal with the challenges in his/her life.

The *Adaptive Play Profile* is the uninterrupted, forward-moving, joyful play activity where the child uses coping strategies like problem solving and anticipation to cope with sources of distress and discomfort in his play activity. Frequently used adaptive coping strategies include a capacity to enjoy the play situation, a capacity for resourceful manipulation and problem solving, and a capacity for representation and symbolization of disturbing experiences and fantasies. The *Inhibited Play Profile* portrays a struggle between unconscious conflicting needs and emotions which create the tensions that find expression through the child’s play activity. Such children have difficulty sustaining free spontaneous play and express a narrow rigid range of affects, with predominant expression of anxiety and an overall somber tone. Coping strategies such as rationalization and isolation of affect are preferred where ideas are separated from their threatening affects. These children usually play alone and silently. The *Impulsive Play Profile* reflects more sharply the conflictual issues and is characterized by an absence of regularity in the flow, marked by outbursts and abrupt interruptions. These children try to cope with disturbing feelings through movement and activity. They rigidly divide the world between the bad and the good because of their difficulty integrating aggressive feelings. Anxiety and the aggression in play are acted out directly in behavior, without translation into symbolic representation. The *Disorganized Play Profile* is characterized by extreme anxiety. At these moments the child is communicating the dread of becoming completely overwhelmed. Such play involves many disruptions and little facilitation on the child’s part. Play activity may involve sensori-motor levels below the child’s developmental age. Play characters and themes contain bizarre and overly aggressive elements and change without the child’s control. Affect may be extreme, predominantly negative and inappropriate, at times involving intense fears of losing control. The child may lose awareness that this is play and feel surprised or frightened by what comes up. These children are unable to use common symbols communicated through the shared use of language.

The profiles reflect a continuum of coping strategies, some more adaptive than others. Children who primarily show characteristics of Adaptive Profile can use play toward gaining mastery by using strategies such as problem-solving, humor and anticipation in play. Children who show characteristics of the other three profiles use strategies that are less effective and use defenses that restrict their capacity to play. Children characteristic of the Inhibited Profile use strategies such as isolation of affect, rationalization and undoing to cope with emotional implications of the play in a neutral, factual, objective way, which results in a rigid play structure. Children characteristic of the Impulsive Profile use movement and activity to distance themselves from threatening experiences and use strategies such as denial and splitting which result in abrupt changes of affect and behavior and disrupt the flow of play. Children characteristic of the Disorganized Profile are unable to use a coping strategy and are overwhelmed by disturbing experiences that can feel traumatic.

In a series of single case studies (Chazan (2000, 2001, 2002), Chari et al. (2013), and Chazan et al. (2016) have shown that the profiles can be used to document changes across therapy with children ranging from ages 2 to 9 and varying levels and types of psychopathology such as Reactive Attachment Disorder, Major Depression, Social Anxiety, Narcissistic Personality Disorder, Borderline Personality Disorder, and Pervasive Developmental Disorder. In each case, the profiles provided a valuable tool for observation and empirical study of play of children with different psychopathologies. This common language for analysis of the play activity provided a systematic lens regarding the therapeutic process. Findings showed that each child started to show more Adaptive Profile characteristics as the treatment progressed. However, an important finding was that the Adaptive Profile was never free of conflict and defense and always existed with the presence of the other profiles. This implies that if the treatment is working effectively, the child uses the play scene to reenact certain problematic situations captured through the lens of Inhibited, Impulsive and/or Disorganized Profiles, however, still uses the play field as part of an overall coping effort to master these problematic scenarios. Thus, there is expected disorganization and displeasure during the treatment phase as children start to bring problematic issues into the play space. It is the completed reparation of the evoked issues that categorizes the treatment process as adaptive. This reparation can only happen through children’s engagement in the play space to enact and work on problematic issues. In order to tap into this process, Chazan (2009) has created an index called Play Engagement that assesses the child’s sustained interest in play over the course of treatment. Chazan (2012) and Chazan et al. (2016) have found that Play Engagement is positively correlated with Adaptive Play and also significantly improves over the course of treatment affording the child the possibility to bring to play different aspects of his internal world.

As the profiles indicate, the act of play is a complex system, containing many different variables describing levels of relationship, levels of cognitive and affective development, as well as capacity for creating narrative. The function of these play profiles is to capture the essence of play activity in all its intricacy. Moreover, the transition between the profiles that happen over the course of treatment involve phases of integration as well as disorganization as the child faces problematic issues which are reworked in the safety of the play sphere. The emergence of different play profiles can hardly be controlled, and predicting at what point in time which aspects of the profiles come to the fore is extremely difficult. In this case, an appropriate methodology that captures the essence of the play activity is needed in order to study the therapeutic process through the lens of play. Quite recently, there have been a number of applications of non-linear dynamic systems perspective to psychodynamic practice with children, which can take into account the various components of the therapy space as well as a non-linear therapeutic action of change which are so central to psychodynamic theories (Coburn, 2002; Sander, 2002; Tyson, 2005; Galatzer-Levy, 2009). The essential characteristics of the theory include the presence of a large number of elements that interact in a dynamic fashion where any element of a given system influences and is influenced by many others. Interactions are also non-linear, which insures that there can be abrupt and unexpected changes. Another main premise of the theory is that for change to happen, there needs to be alternation between stable states and disorganization through which new and more flexible forms of functioning can emerge (Coburn, 2002).

Recognizing the importance of discontinuous changes in psychotherapy process, some authors have included the theories of non-linear systems and self-organization in their concepts of psychotherapeutic change (Tschacher et al., 2000; Gumz et al., 2013; Schiepek et al., 2014; de Felice and Andreassi, 2015; Orsucci, 2015) combining psychodynamic considerations and a self-organization theory of synergetics. The general idea here is to treat the therapeutic space as a complex system that may be characterized by processes of pattern formation with stable and unstable episodes (i.e., phases in which the system elements fluctuate to varying degrees) and abrupt transitions. In terms of the dynamic systems theory, a pattern which characterizes the behavior of a dynamic system over a period of time in a relatively stable manner is referred to as attractor in the sense of an attractive dynamic state. If we consider the play profiles of the children, we can regard the emerging “profiles of play” as attractors (stable states). In other words, a specific play profile dominates the psychotherapy field until the child is ready for shifting toward a new organization. A transition from one stable play profile to another can occur via temporary destabilization of the system. Such destabilization occurs when the energy level of the system is changed. If a certain energy level is reached, that is, if a certain control-parameter limit has been exceeded, increasing instability leads to critical fluctuations. In such cases, system behavior can abruptly switch to a new pattern of behavior. During that period of fluctuations, the system is open for processing new information, ready to explore potentially more suitable configurations. There is, at this stage, an alternation between older and less functional forms of internal organization and new, emerging configurations.

### Aims of the Current Study

In the present study, the processes of change were investigated through the lens of play activity, in three single case long term psychodynamic treatments, by comparing changes in play profiles as the therapy progresses. In accordance with specified criteria for replicated single-case design, a small set of successive cases with the similar demographic characteristic and the same presenting diagnosis were examined (Ollendick et al., 2006). Single case research has often been indicated as one of the most suitable approach for evaluating psychodynamic process and used effectively in process and outcome research (e.g., Hilliard, 1993; Orlinsky et al., 2004). The cases were three 6-year-old girls with a presenting problem of Separation Anxiety. This age group and the presenting problem are amenable to study change processes in psychodynamic child psychotherapy as documented in a recent review by Midgley and Kennedy (2011) who found that younger children appear to benefit more from psychodynamic psychotherapy than older ones, with the likelihood of improvement during treatment declining with age and children with Internalizing Disorders, especially with anxiety and depression, seem to respond better than those with disruptive/Externalizing Disorders.

The main research questions investigated are: (a) whether there is a reconfiguration in the children’s internal world operationalized as a change in the CPTI play profiles over the course of treatment; (b) whether there is emergence of new and more functional organizations of CPTI play profiles in the second phase of the treatment. In case these questions are verified, the indices predicting the change of the children’s internal world are also investigated. For these purposes, the children’s play profiles were calculated by the CPTI coding system taking into account four different play profiles, Adaptive, Inhibited, Impulsive, and Disorganized. Moreover, in order to understand more specifically how the children used the play space, their level of “Play Engagement,” the “Play Themes,” and “Relational Themes in Play” were also taken into account. These variables were clustered and their development was studied with the aim of understanding the evolution of profiles in the three treatments by non-linear time series analyses such as Markov Transition Matrix, Recurrence Quantification Analysis (RQA) and odds ratios comparing the first and second halves of psychotherapy.

## Materials and Methods

### Participant Selection and Description

The patient data came from the Istanbul Bilgi University Psychotherapy Research Laboratory, established in order to study the psychotherapy processes conducted at Istanbul Bilgi University Psychological Center, which provides outpatient psychodynamic psychotherapy and professional training at master’s level for students in the Clinical Psychology Program. Participants were not recruited for participation in a research study, in an effort to increase generalizability and limit sample-selection bias. Further, it was the intention of the researchers to examine the process of psychodynamic psychotherapy with children commonly encountered in real-world clinical settings, which is in contrast to a highly controlled sample, typically sought in treatment-outcome studies.

Sample selection occurred following completion of study planning and was based upon the following inclusion criteria: ages between 4 and 10 years old; average intelligence; motivation for treatment; no psychotic symptoms; no significant developmental delays; no significant risk of suicide attempts; no drug abuse. The children of the three families included in this study were three Turkish females, all 6 years old. All children were brought to therapy because of anxiety due to separating from their mothers and problems with school attendance. All families were from a middle socioeconomic status (mid SES) composed of married biological parents. Each of the children had at least one sibling. The educational level of the parents included a graduate equivalency diploma. None of the children had previously been in psychotherapy. The parents provided written informed consent and the child provided oral assent concerning use of their data for research purposes. This research was approved by Istanbul Bilgi University Ethics Committee.

### Treatment Integrity and Outcome

The therapists, therapists’ training, and supervision. All patients had different therapists, who were female, master’s level clinicians with 1–2 years of professional practice experience. Formal training included theoretical background of psychodynamic play therapy and its various applications 1 year prior to the study. All therapists were supervised by experienced psychodynamic play therapists during the study. In this way, the confounding variables rooted in differences in the educational background, experience, and supervision process were partially controlled.

#### Treatment

The treatment was psychodynamic play therapy. The treatment was not manualized and the only restrictions placed were regularity and length (once weekly treatment of 50 min for 1 year). Patient 1^1^ (Rengin) received 59, patient 2 (Esin) received 55 and patient 3 (Canan) received 47 sessions. Even though there is no unitary model of therapeutic action in psychodynamic play therapy (Fonagy, 2004), the core principles and techniques employed can be summarized as follows: central to this approach is the establishment of what is called a “setting.” The psychotherapist sees the child at regular times, in the same play room with a standard set of play toys. This consistency provides a safe context that allows the child to play out difficult and disturbing emotional experiences that would be hard to express in the outside world. The exploration of the child’s issues takes place in a largely child-led process way and the therapist encourages the child to express and reflect on his perceptions, feelings and thoughts in play. This is done by listening actively and inviting the child to continue his communications and asking questions about the play setting, temporal ordering, and the details of the characters, their thoughts, feelings and behaviors. Interpretations aim to help the child see links between conflicting needs and emotions about self and others that find reflection in play behaviors and in the therapeutic relationship with the purpose of bringing to consciousness attitudes, assumptions and beliefs of which the child is unaware.

#### Assessment of Psychotherapy Outcome

Overall, each child presented with separation anxiety problems and demonstrated marked reduction in symptoms during treatment. At initial assessment interviews, each child’s scores on The Child Behavior Checklist (CBCL; Achenbach, 1991; Erol et al., 1995), a widely used method of identifying problematic behaviors in children, were at the borderline or clinical level for “Internalizing Disorders” and “Anxiety Problems” on CBCL’s Diagnostic Statistical Manual (DSM) oriented Scales, indicating the need for treatment (see **Table 1**). On the CBCL, the clinical cut-off for the “Internalizing” subscale is 64 and for “Anxiety Problems” the clinical cut-off is 70. According to Jacobson and Truax (1991) the “Reliable and Clinically Significant Improvement” was used as a measure of change over the two time-points. Reliable Change Index (RCI) for CBCL and The Children’s Global Assessment Scale(CGAS; Shaffer et al., 1983) a numeric scale (from 1 to 100) used by mental health clinicians to rate the general functioning of children, were calculated as improved if the patient’s scores at termination, when subtracted from scores at admission, and divided by the standard error of the instrument were above 1.96. Norm data for the CBCL and CGAS were used to assess individual scores in relation to clinically significant change. Notably, RCI indicated clinically significant change from pre to post-treatment on relevant CBCL subscales (see **Table 1**). For CGAS each child’s scores on global functioning fell within the good functioning range at post-treatment and RCI was significant. Given that the patients crossed the limit from clinical to normal population and this change is not attributable to measurement error, we concluded that changes in scores are clinically significant. Having demonstrated that these were ‘good outcome’ cases, the nature of the change across the course of therapy was investigated using the CPTI.

**Table 1:** Reliable change indices.

	Pre-treatment	Termination	RCI > 1,96
**Patient 1 (Rengin)**			
CBCL Internalizing Problems	**63**	50	4,64^∗^
CBCL Anxiety Problems	***65***	57	6,67^∗^
CGAS	56	78	4,26^∗^
**Patient 2 (Esin)**			
CBCL Internalizing Problems	*67*	46	7,05^∗^
CBCL Anxiety Problems	**66**	50	13.33^∗^
CGAS	51	75	4,65^∗^
**Patient 3 (Canan)**			
CBCL Internalizing Problems	**61**	45	5.71^∗^
CBCL Anxiety Problems	**64**	50	11.66^∗^
CGAS	50	79	5,16^∗^

*Bolded and italicized scores indicate, respectively, borderline and clinically significant scores. ^∗^RCI significant. CBCL, Child Behavior Checklist; CGAS, Children’s Global Assessment Scale; RCI, Reliable change index.*

### Instruments and Play Profile Calculations

#### Assessment of Play Activity

Children’s Play Therapy Instrument (CPTI; Kernberg et al., 1998) rates children’s behavior in a therapeutic setting at different levels (see **Figure 1**; for further definition of play activity categories, see Chazan, 2000, 2001, 2002; Chazan and Wolf, 2002). The play profiles are calculated in three steps. The first step involves a “*Segmentation* of the child’s activity” (non-play, pre-play, play and interruption). Going forward, *only play segments* are rated.

Once the play segment has been identified, the second step involves rating the play segment on individual CPTI items. These CPTI items all belong to a particular component, listed under different theoretical levels of analyses (i.e., descriptive, structural, and functional), defining an aspect of play activity. Each of these items is scored using a 5-point Likert scale: 5 = Most Characteristic; 4 = Considerable Evidence; 3 = Moderate Evidence; 2 = Minimal Evidence; 1 = No Evidence. In order to provide an example of scoring, in the play sphere component, under descriptive analysis, there are three items: autosphere, microsphere, and macrosphere. Each item is assigned a score from 1 to 5, answering the question “does the child use actual space of the room?” (macrosphere), “does the child play in the miniature toy word?” (microsphere), “does the child play with reference to his body?” (autosphere). The same process takes place for each item of the scale. After all these items are scored, the profiles are calculated manually by taking an arithmetic average of particular items associated with each profile (see calculation of profiles below). Before explaining the calculation of profiles, we will provide a description of each play component

1. The *Descriptive Analysis* includes components that describe the play observed:
  1a. Script Component of Play Activity: This components looks at the contribution of the child to the unfolding of play activity.
  1b. Sphere Component of Play Activity: This component looks at the spatial realm within which the play takes place taking into account whether the child plays with reference to his body, in the realm of miniature toys or using the actual space of the room.
2. The *Structural Analysis* is comprised of the underlying processes necessary for the formation of characters and the telling of a story and uses the following four components:
  2a. Affective Component. This component looks at the types, range, and regulation of emotions brought by the child to play.
  2b. Cognitive Component. This component looks at the level of role play, specifically Complex, Dyadic, and Solitary Roles, and how persons and objects are depicted, specifically Realistic, Magical, and Bizarre Representations, and if Transformations occur in the way persons, toys, and other objects are used. It is critical to note if these transformations of characters, or objects, occur unexpectedly.
  2c. Language Component. This component looks at the kinds of language used by the child.
  2d. Social Component. The social level of play indicates interaction of the child playing with the therapist.
  2e. Narrative Component. This component looks at the relational themes of the interactions (i.e., autonomous, dependent, malevolent control) and play themes.
3. The *Functional Analysis* of the child’s play activity is used to observe coping/defensive strategies of the child.
  3a. Coping and Defensive Strategies Component is grouped along a continuum into four items: *Defense Cluster 1* (Adaptive, i.e., adaptation, problem-solving, sublimation, humor), *Defense Cluster 2* (Conflicted, i.e., intellectualization, doing and undoing, somatization, avoidance), *Defense Cluster 3* (Polarized, i.e., splitting, projective identification, omnipotent control), *Defense Cluster 4* (Extreme Anxiety, i.e., dispersal, fusion, dedifferentiation, autistic encapsulation, freezing).
  3b. The Child’s Awareness of Himself as Player indicates his awareness of being in a state of play.

#### Calculation of Play Profiles

As per the manual, four clusters of Profiles were calculated: the Adaptive Profile; the Impulsive Profile; the Inhibited Profile; and the Disorganized Profile. In addition another index that reflects the child’s overall “Play Engagement” was used in order to reflect the child’s overall investment in play. These profiles are calculated by calculating a composite score using each CPTI item that is associated with this profile (see **Table 2**). In order to create the composite profile scores, all the specific items under each profile were summed and then divided by the number of items in that category. Because all the items are scored on a scale of 1–5, a standardization procedure is not needed. These overall scores are used for each profile. The internal consistency of the profiles was tested by Cronbach alpha and the scores varied between 0.66 and 0.77 indicating good reliability.

**Table 2:** Children’s Play Therapy Instrument (CPTI) Profile Items.

CPTI Categories	Adaptive Profile	Inhibited Profile	Impulsive Profile	Disorganized Profile	Play Engagement
Script Description	Facilitate Play	Inhibition	Inhibition	Inhibition	Facilitate Play
Play Sphere	Microsphere		Macrosphere		
Affective Components	Flexible Affect Regulation	Rigid Affect Regulation	Rigid Affect Regulation	Rigid Affect Regulation	Flexible Affect Regulation
	Smooth Affect Transitions		Abrupt Affect Transitions		Smooth Affect Transitions
	Pleasurable Tone	Somber Tone	Overt Distress	Overt Distress	Pleasurable Tone
			Anger Expression	Anger Expression	
		Narrow Affect Spectrum			
	Appropriate Affect to Content			Inappropriate Affect To Content	
Cognitive Components	At Least one Play Role	Solitary Play Roles			
		Self-Related Relations			
	Voluntary Transformation of Play Roles		Involuntary and Unstable Transformations	Involuntary and Unstable Transformations	
			Magical Play Roles	Bizarre Play Roles	
Language Components	Verbalizing Characters	Silence			
	Describing Play				
Social Components		Play Alone			
Coping and Defensive Strategies	Adaptive	Conflicted	Polarized	Extreme Anxiety	Adaptive
Awareness of Being in a State of Play	Aware		Unaware	Unaware	

The specific CPTI items that contribute toward each Profile are as follows:

##### Adaptive profile

Facilitation of Play, Play in Microsphere, Regulation of Affects: Flexible, Smooth Transitions between Affects, Hedonic Affective Tone: Pleasurable, Appropriate Affect Expression to Play Content, One or More Play Roles, Voluntary Transformation of Play Roles, Talking about the Play (Verbalization of Roles, Describing the Play, Defense Cluster 1: Adaptive Strategies, Awareness of Being in a State of Play.

##### Inhibited profile

Inhibition of Play, Hedonic Tone: Somber, Spectrum of Affects: Narrow, Regulation of Affects: Rigid, Solitary Play Roles, Level of Relationship: Self-Related, Use of Language: Silence, Play Alone, Defense Cluster 2: Conflicted Strategies.

##### Impulsive profile

Inhibition of Play, Play in Macrosphere, Affective Tone: Overt Distress, Regulation of Affect: Rigid, Abrupt Transitions Between Affects, Affect Type: Anger, Involuntary and Fluid Transformation of Play Roles, Magical Representations, Defense Cluster 3: Polarized Strategies, Unaware of Being in a State of Play.

##### Disorganized profile

Inhibition of Play, Affective Tone: Overt Distress, Inappropriate of Affect to Content, Affect Expressed: Anger; Involuntary and Fluid Transformation of Play Roles, Bizarre Representations in Play, Isolated Play, Defense Cluster 4: Extreme Anxiety Strategies, Unaware of Being in a State of Play.

##### Play engagement

Facilitation of Play, Hedonic Affective Tone: Pleasurable, Regulation of Affects: Flexible, Smooth Transitions between Affects, Defense Cluster: 1 Adaptive Strategies.

The first author was trained by Saralea Chazan on the use and adaptation of CPTI. The CPTI was translated and back translated for use in Turkey. A group of seven graduate students and an experienced clinical psychologist with 10 years of clinical experience evaluated the language and statement comprehensibility of the scale. The scale was finalized following necessary modifications according to the feedback received during this evaluation. Two masters level clinical psychology students who received 20 h of training on the CPTI by the first author and rated 10 training sessions (24 play segments) prior to the study rated the sessions. They were independent assessors who were not associated with the treating clinicians or the cases, and blind to the purposes of the study. In order to identify the agreement level between judges for this current study, we calculated the Interclass Correlation Coefficient (ICC) for ordinal variables which ranged from good to excellent (ICC = 0.78–0.89).

#### Coding and Ratings of Sessions

All the sessions were videotaped and transcribed verbatim. Using the CPTI, the all the sessions were segmented. The number of segments for a session ranged from 4 to 7 (*M* = 5, *SD* = 1.3). These segments were chunked into four categories: Pre-play, Play Activity, Non-play, and Play Interruption. As per CPTI coding manual, the longest Play Activity segment within a session was used for further analyses.

#### Data Analytic Strategy

The CPTI coding of play segments generated four main profiles: Adaptive, Impulsive, Inhibited, and Disorganized. In order to have as much information as possible regarding the child’s use of the play space, three other indices were included into the analyses: The children’s “Play Engagement,” the two most characteristic “Play Themes” and “Relational Themes in Play.” The rationale for data analysis can be summarized as follows: Our goal was to quantify play dynamics of each patient and study its evolution. In order to do so we tested whether the descriptors we use to codify play activity have the same meaning for all the patients. This was made by checking the invariance between descriptors’ correlation structure (Pearson correlation coefficient) across different patients. The three cases showed the same correlation structures across treatment for play profiles as demonstrated by their common negative correlation between Play Engagement and Disorganized and Inhibited Profiles (-0.86 < *r* < -0.63, *p* < 0.01); positive correlation between Disorganized, Impulsive and Inhibited Profiles (0.46 < *r* < 0.62, *p* < 0.01); and a positive correlation between Adaptive Play and Play Engagement (0.51 < *r* < 0.62, *p* < 0.01) making them susceptible to cross-comparisons. Afterward, we looked for ‘discrete states’ in terms of clusters that can describe the entire psychotherapy space through *K*-means cluster analysis. In this analysis, each cluster corresponds to a specific average profile in the descriptor space. The trajectories between clusters were analyzed through Markov Transition Matrix, which can be thought as consecutive transitions in which at each discrete time point, the system decides to remain in the same cluster or shift to another one. This also tests for the ergodicity of the system answering whether the child can reach any state or shift to another one in play. We also checked for change in state probabilities among the first and second halves of the therapy. RQA is a widely used non-linear time series analysis tool that builds upon the repetitions (recurrences) of the same state in time, thus it is perfectly suited to investigate such volatility in the series in psychotherapy process research.

## Results

### Clusterization of Play Variables

Firstly, in order to understand how these different variables are grouped together, a clusterization procedure was conducted. With the aim of finding the best cluster solution, we took the first peak of explained variance followed by a decrease or at least a stationary value. The first peak followed by a stationary value corresponded to the seven clusters solution, explaining the 74% of variance. Consequently we performed a *K*-means clusterization on the dataset with the seven cluster solution. Each cluster corresponds to a specific pattern of the play variables. We called these specific patterns “states” of the complex system composed of our three single cases’ play profiles and play characteristics during each play segment. (see **Tables 3** and **4**).

**Table 3:** Cluster Table of CPTI variables.

Final Cluster Centers							
	Clusters						
	1	2	3	4	5	6	7
Play Engagement	3.55	3.76	3.71	3.79	3.90	3.51	3.90
Adaptive Play	2.70	3.08	2.91	3.17	3.03	2.86	3.16
Inhibited Play	2.18	2.02	2.04	1.94	1.91	2.08	1.90
Impulsive Play	2.30	1.97	2.00	2.55	1.68	2.00	2.30
Disorganized Play	1.98	1.78	1.85	2.04	1.61	1.89	1.82
Narrative Theme (Most characteristic)	3	2	6	11	5	2	3
Narrative Theme (Second most characteristic)	3	6	2	3	8	2	3
Relational Theme (Most Characteristic)	6	2	2	2	2	2	2
Relational Theme (Second most characteristic)	4	4	4	3	4	4	3

*The specific patterns between variables describe their cluster membership.*

**Table 4:** Cluster Membership of 3 patients’ Play Segments.

Cluster Membership			
Play Segments	Patient 1 (Rengin) Cluster Memberships	Patient 2 (Esin) Cluster Memberships	Patient 3 (Canan) Cluster Memberships
1	3	2	6
2	6	2	6
3	3	2	2
4	3	3	5
5	2	6	3
6	6	2	6
7	2	2	6
8	3	6	2
9	5	3	7
10	5	2	6
11	7	5	6
12	4	7	2
13	2	2	6
14	2	2	6
15	2	5	6
16	3	2	2
17	2	1	2
18	7	5	2
19	3	2	5
20	3	5	3
21	5	6	2
22	2	6	2
23	2	6	5
24	5	1	3
25	6	1	6
26	6	7	2
27	2	3	3
28	2	6	3
29	5	2	3
30	2	2	7
31	2	6	7
32	2	2	2
33	6	2	3
34	5	6	2
35	2	1	2
36	2	6	6
37	6	3	3
38	2	2	7
39	6	6	6
40	5	2	7
41	2	6	4
42	7	7	4
43	6	2	3
44	6	6	3
45	3	5	7
46	4	5	4
47	2	6	3
48	3	2	
49	7	3	
50	2	3	
51	3	6	
52	4	1	
53	3	3	
54	3	1	
55	5	6	
56	3	6	
57	5	5	
58	3		
59	5		

### Clinical Description of Each Cluster

In the first cluster, the children show their lowest scores for Adaptive Profile (2.70) and Play Engagement (3.55) and highest scores for Inhibited Profile (2.18). This kind of play is typically somber in tone, characterized by rigidity and the expression of anxiety. There are no significant interactions with others or imagined roles portrayed. The predominant narrative themes in this cluster centered about issues around cleanliness. In the second cluster, the children’s Adaptive Profile (3.08) and Play Engagement Characteristics (3.76) are at their second highest value and the other profiles are relatively suppressed. This kind of use of the play space show initiation, facilitation and shifting of play activity; overall expression of pleasant flexible emotions, multiple characters in play under the creative control of the child, descriptive language of play activity and higher levels of social development involving reciprocity and cooperation. Play themes revolve around attachment and issues regarding affection and nurturance as well as danger and protection. The child is primarily showing dependent relational qualities in play. In the third cluster, the children’s profile scores are relatively suppressed compared to other clusters with play themes again having to do with separation, attachment, danger/protection and nurturance. It is possible that the children are externalizing their issues without significant coping strategies and just using the play space as a scene to put forth their concerns. In the fourth cluster, the children show highest scores for Adaptive Profile (3.17), Impulsive Profile (2.55) and Disorganized Profile (2.04) and scores for Play Engagement (3.79) are at their second highest value. The play themes revolve around aggression and cleanliness. The dominant relational quality among play characters is again dependent. It is possible to surmise that the adaptive component helps the child show disorganized and impulsive strategies without a significant rupture in their play engagement and thus try out less adaptive strategies in the safety of the play space. In the fifth cluster, the child shows similar characteristics as in the second cluster, most predominantly showing adaptive strategies and suppressed scores for other profiles with the highest scores for Play Engagement (3.90). There are new themes in play having to do with competition and construction indicative of the child’s ability to expand the play narrative. In Cluster 6, the children’s Play Engagement (3.51) scores are at their lowest value, and Adaptive Profile (2.86) is at its second lowest value whereas Inhibited Play (2.08) is at its second highest. This kind of play is again indicative of rigidity, conflict and anxiety as in Cluster 1, with play themes focused on issues regarding attachment and separation. The children are precluding mutual social interactions and showing primarily dependent relational qualities. Finally, Cluster 7 shows relatively high scores for Adaptive Profile (3.16) as well as Play Engagement (3.90) with also Impulsive Strategies at their second highest value (2.30). Play themes revolve around attachment, however, there are also issues regarding aggression. There is an adaptive use of the play space, however, the children also show impulsive strategies characterized by an absence of regularity in the flow and modulation of the play activity which may be marked by outbursts and abrupt interruptions. This style of playing is linked with expression of feelings through movement and activity.

### Markov Transition Matrix

**FIGURE 2:**
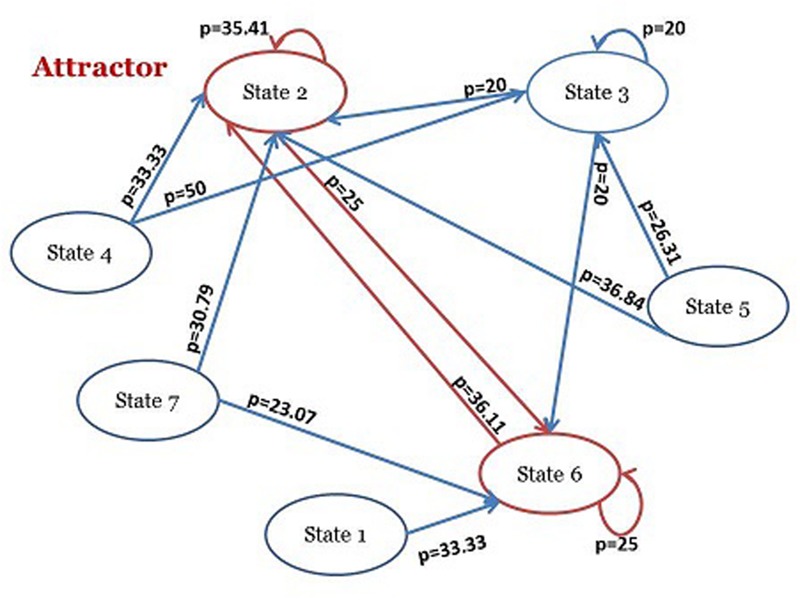
**Simplified version of the Markovian Visual Representation**.

The trajectories between these states can be thought as consecutive transitions in which at each discrete time point the system decides to remain in the same cluster or shift to another one. This kind of dynamics can be formalized in terms of first order Markov chains (or transition matrixes) reporting as rows the states at time *t*, and as columns the state at time t+1. Each i, j element of a Markov Transition Matrix reports the relative frequency of a single step transition from state i to state j across the entire series. The transition matrix of the studied series is performed according to the above description. Visually we could represent the transition matrix with a diagram through which the main features of this complex system composed of the children’s psychic states are shown (**Table 5**; **Figure 2**). The transition matrix of the three single cases shows the emergence of an attractor composed of the states 2 and 6. This oscillation represents the most frequent dynamic in terms of probability of this system.

**Table 5:** Markov Transition Matrix.

Clusters	1	2	3	4	5	6	7
1	16.66	0.0	16.66	0.0	16.66	33.33	16.66
2	2.08	35.41	16.66	0.0	14.58	25	6.25
3	3.33	20	20	6.66	16.66	20	13.33
4	0.0	33.33	50	16.66	0.0	0.0	0.0
5	0.0	36.84	26.31	0.0	10.52	15.78	10.52
6	8.33	36.11	13.88	0.0	11.11	25	5.55
7	0.0	30.79	15.38	23.07	0.0	23.07	7.69

*Clusters represent the transition probabilities between different states (i.e., the child’s ways of using the play activity).*

### Recurrence Quantification Analysis (RQA)

The sequences of seven states can be turned into a still more basic binary series by putting a 0 every time the trajectory does not change state and a 1 when it changes (independently of the particular state). These binary series are strictly related to the temporal evolution of the system variability: long sequences of ‘zeros’ point to low variability (or volatility or entropy), on the contrary long sequences of ‘ones’ to extremely variable regimes. RQA is a widely used non-linear time series analysis tool invented with the aim of studying the repetitions (i.e., recurrences) of the same symbol (i.e., state) over time, thus it is perfectly suited to investigate the above mentioned variability. Therefore, we compared the Recurrence and Determinism values of the three time series with 30 artificially generated randomly shuﬄed series. The Recurrence and Determinism values for the real time series were 0.626 and 0.462, whereas the shuﬄed series were 0.626 and 0.503, respectively, which shows that there was no difference in terms of recurrence between the shuﬄed series and the real ones. This means that the three single cases don’t have a specific trend. They stay or leave a given state in a given period of time with the same frequency as a randomly shuﬄed series does. Given that there is no trend within the three single cases we further investigated what the transitions depend on.

### Ergodicity Analysis

Ergodicity checks whether the time spent by a system in some region of the phase space is proportional to the area of this region. In our case that is to say that the probability of transition from the state I to state j is proportional to the relative frequency of state j. Given the relative ‘distance’ between the states (how big their profile differences are) does not influence the transition probability, this implies the entire phase space is ‘reachable’ by any position. We found in fact that the very high Pearson Correlations between the composite frequency and number of transition reveal that any point of the entire phase space can be reached by any other point of the same space (**Table 6**). In fact, the number of transitions scale almost perfectly (*r* = 0.91, *p* < 0.01) with the composite frequency and the correlation remains high even if we eliminate the effect of distance (partial *r* = 0.89). This implies that the entire phase space is ‘reachable’ by any position. So in terms of the child’s different ways of using the play space, according to our data set, no stable configuration took place and the situation is still fluid and reversible. However, whether and how the psychic system of each of the three patients is evolving over time still needs to be tested.

**Table 6:** Correlation Matrix between the Number of Transitions and Composite Frequency.

Correlation Matrix	Distance	Number of Transition	Frequency State “I”	Frequency State “J”	Composite Frequency
Distance	1.00	-0.47^∗∗^	-0.34^∗^	-0.34^∗^	-0.48^∗∗^
Number of Transitions		1.00	0.51^∗∗^	0.51^∗∗^	0.91^∗∗^
Frequency State “I”			1.00	-0.16	0.57^∗^
Frequency State “J”				1.00	0.57^∗^
Composite Frequency					1.00

*^∗^Correlation is significant at the 0.05 level; ^∗∗^Correlation is significant at the 0.01 level.*

### Evolution of Play Profiles Over Time: Odds Ratios

Some states could be more frequent in the late phase (second half of the therapy), and others could be more frequent in the initial phase (first half of the therapy). We investigated the evolution dynamics of each state counting the number of occurrences in the first and in the second half of the therapy in terms of odds ratio (**Table 7**). The results showed a slight decrease in the occurrence of clusters 7 and 6 for patient 2 (Esin) and patient 3 (Canan) respectively. Patient 1 (Rengin) instead, did not show any changing trend but probably acquired awareness of her troubling internal states by experiencing them within the play segments. We tested this further hypothesis by calculating the odds ratios of time spent in play, non-play, pre-play, and interruptions for each patient. We expected that Rengin increased the number of play segments in the second half of treatment. This hypothesis was partially verified (**Table 8**). Rengin decreased the number of interruptions during play over the course of the second half of treatment to some extent, an index that seems related to the acquired competence to deal with troubling internal states in play.

**Table 7:** Odds Ratios of Each Cluster per Patient in the First and Second Halves of Therapy.

	Patient 1 (Rengin) Odds Ratio	Patient 2 (Esin) Odds Ratio	Patient 3 (Canan) Odds Ratio
Cluster 1	n.d.	1.03	n.d.
Cluster 2	1.14	1.298	2.09
Cluster 3	1.03	0.781	0.229
Cluster 4	0.515	n.d.	n.d.
Cluster 5	1.03	1.37	n.d.
Cluster 6	0.825	0.622	3.128 *1.82ES*
Cluster 7	1.03	2.08 *1.61ES*	0.206
Mean	0.795	1.025	0.807
*SD*	0.407	0.655	1.270

*n.d., non determined (i.e., the denominator of the ratio is equal to 0). Italics show the standard errors from the mean. The analysis does not show any statistically significant *p*-values (95% CI).*

**Table 8:** Odds Ratios of Type of Activity per Patient in the First and Second Halves of Therapy.

	Patient 1 (Rengin) Odds Ratio	Patient 2 (Esin) Odds Ratio	Patient 3 (Canan) Odds Ratio
Non-Play	0.809	0.513	0.714
Pre-Play	1.083	0.56	1.555
Play	1.034	1.65	0.958
Interruptions	*21.48ES*	1.32	0.5
Mean	1.23	1.01	0.93
*SD*	0.52	0.56	0.45

*Italics show the standard errors from the mean. The analysis does not show any statistically significant *p*-values (95% CI).*

## Discussion

This paper proposed a novel model to study change in psychodynamic play therapy looking at the play field as a complex system. The processes of change were assessed through a repeated systematic assessment of three children’s play profiles, and their evolution over time. Our research questions were partially confirmed. We found a change in terms of the reduction in the occurrence of Clusters 6 and 7, however, we have not seen the emergence of a new play profile. The results showed two distinct mechanisms of change that can illuminate the process and trajectory of improvement: The emergence and expression of the children’s psychic states in play, as shown in the ergodicity of the system, which implies that the child can move between different states at any given point in time to express and work on different aspects of his internal experience in play; and the early process of modification of less dysfunctional states in play, as demonstrated in decrease of some less adaptive profiles and a decrease in play interruptions.

### Evolution of Play Profiles

The first research question had to do with investigating whether there is a reconfiguration in the children’s internal world operationalized as a change in the CPTI play profiles, play and relational themes over the course of treatment. Instead of a definitive reconfiguration, we found that children used psychotherapy to express different psychic states associated with their presenting problems. The results showed that the oscillation between Clusters 6 and 2 were the most frequent dynamic of the treatments. The clinical content associated with each cluster showed that Cluster 2 had to do with the predominance of the Adaptive Profile and Play Engagement, whereas Cluster 6 had to with Inhibited Profile and low levels of Adaptive Play and Play Engagement. The fact that the children expressed similar play and relational themes in Clusters 2 and 6, mainly having to do with issues regarding attachment, separation, danger, and protection, shows that these clusters represent two different coping strategies for similar play narratives. Children with Separation Anxiety are known to suffer from excessive fear and distress concerning separation from home or significant attachment figures, worrying about their own or their parents’ safety. The play themes are reflective of these concerns, and in Cluster 6, create significant inhibition and conflict. This is consistent with literature that shows that anxious children have been shown to play solitary, engage less readily in dyadic play and are more inhibited in play (Christian et al., 2011). However, children in our study are oscillating between Inhibited (Cluster 6) and Adaptive Profiles (Cluster 2) in face of the emerging issues, which parallels prior findings by (Chazan, 2002; for a review), who showed that children use the play scene to reenact certain problematic situations which can cause temporary stagnation, however, as long as the child continues to play symbolically, adaptive strategies are also used as part of an overall coping effort to master these problematic scenarios.

Play activity is an avenue for expressing feelings and thoughts without fear of reprisal or irreversible outcome. The realm of play activity introduces an “as if” quality, allowing for freedom of imagination and trial efforts toward mastery (Winnicott, 1971; Fonagy and Target, 1998). This was also shown in the ergodicity of the system indicative of the use the play field as a free space to bring to front internal states uncommonly expressed in usual relationships and provide an opportunity to manage them. In fact, prior studies have shown that the mechanism of play therapy in facilitates mastery through re-enactment of stressful experiences (Singer and Singer, 1990; Gaensbauer and Siegel, 1995; Russ, 2004). Another implication of the child’s oscillations between Clusters 2 and 6 is that Adaptive Profile rarely exist independently of other strategies. In cases where conflict is too much to bear, a defense mechanism may help the child cope, by dividing an emotionally difficult situation into manageable parts or suppressing the excessive threat and helping the child focus on what can be mastered (Chazan, 2002).

The second research question was whether there is emergence of new and more functional organizations of CPTI play profiles in the second phase of the treatment. In these three therapies, a definitive reorganization of the children’s internal world was not observed. Instead, in the final stage of the year, there was an initial phase modification of previously dysfunctional states. This latest process concerned the decrease of Clusters 7 and 6 for patients 2 (Esin) and 3 (Canan) respectively. Patient 1 (Rengin) instead did not show any changing trend in terms of clusters but showed a decrease in the number of interruptions in play in the second half of treatment. As mentioned before, Cluster 6 has to do with inhibition and conflict associated with issues having to do with separation and danger that the child cannot resolve. Cluster 7, though having significant adaptive qualities, also has relatively dominant impulsive strategies associated with sensori-motor play where feelings, especially anger are expressed through movement and activity. The decrease in both clusters is showing the beginning of the modification of more dysfunctional profiles (i.e., Inhibited and Impulsive Profiles). Patient 1 (Rengin) seems “delayed” in comparison to the others, still dealing with the problem of placing and experiencing some internal states (probably painful) in the play space without observable modification. This is also underlined by Patient 1’s higher percentage of non-play segments (30%) in comparison to Patient 2 (24%) and 3(24%) over the course of treatment, possibly indicating her difficulty in experiencing and managing conflictual internal states in play. However, the decrease in interruptions during play over the course of the second half of treatment is an index of the growth in child’s capacity to play unobtrusively (Chazan, 2000, 2001, 2002). The decrease in play interruptions is indicative of play that is satisfying and fulfilling and experienced by the child as pleasurable and affords her the opportunity to express a variety of affects (Chazan, 2002). These are all positive prognostic signs that Patient 1 has started to contain and regulate her conflicts and associated affects within the psychotherapeutic play field. It is possible that continuing the psychotherapy would afford new patterns of psychic states to come to the surface.

Our third research question was to explore the indices predicting the good outcome of these cases. In these three treatments, our analyses show that symptomatic improvement has to do primarily with the ergodicity of the therapeutic system. When psychotherapy becomes ergodic, the patients are able to bring different aspects of troubling internal states as well as a wide repertoire of coping mechanisms to treatment and test their effectiveness in the play space. This finding parallels recent developments in psychodynamic play literature that has de-emphasized the role of interpretation as a curative factor and instead focused on broadening the child’s self-experience by increasing the range, depth and emotional richness of play (Target et al., 2005). The task of the therapist then is primarily to help the child play and support the child by facilitating a coherent narrative in play, help imagine the inner lives of the play characters and work out conflictual emotional scenarios (Target et al., 2005). Our findings support that this is a necessary step toward the modification of some dysfunctional and/or painful patterns. We were able to witness the initial stages of this process in the reduction of Clusters 6 and 7 having to do with more Inhibited and Impulsive strategies.

### Implications for Psychodynamic Play Therapy Research

Even though there is substantial evidence that play based therapies produce significant change the specific links between play processes and treatment outcome remain unexamined (Shirk and Burwell, 2010). However, many psychotherapists report that in order to deliver more effective therapies the curative factors in play need to be empirically identified (Kazdin, 2003). Our findings support and bring additional findings on the central role of play in psychodynamic treatment process. The initial findings having to do with the predominance of the Inhibited Profile is in line with literature that shows that anxious children have difficulty engaging readily in play (Christian et al., 2011). They tend to remain centered upon themselves and bring solitary representations to the play field (Barnett and Storm, 1981) which are all captured under the items loading toward Inhibited Profile. However, our findings suggest that the treatment affords them the opportunity to assume a different role, other than their own, and proceed to activity in terms of reciprocal interactions as shown in the decrease in Inhibited Profile and the expression of Adaptive characteristics captured by Cluster 2. The oscillations between inhibition and adaptation parallel Chazan’s(2002) findings who showed that children still continue to use less adaptive strategies at the end of the treatment, however, these mechanisms become counterbalanced, and appear in more adaptive, sublimated, playful ways.

In terms of the mechanisms of change, our findings point to the importance of oscillations between different states (as shown in the oscillations between States 6 and 2) and the ergodicity of the system which give the patient the possibility and flexibility to move between different states at any given point. These oscillations afford the child the opportunity to try out new strategies, revert to old ones when their stress is too high which help restore stability toward generating something new. This in line with principles of complex systems that oscillate between disorder and order, instability and the self-regulating processes that restore stability. It is only after disorganization, oscillations and recurrence of previous states that the system generates something new in the psychotherapeutic space (Tyson, 2005; Galatzer-Levy, 2009). Even though we have not seen a definitive reorganization toward a new profile in our data, the decrease in play interruptions as well as the use of less adaptive profiles (Inhibited and Impulsive) are indicative of early phases of change. In fact, Chazan (2002) in her study with an inhibited boy found most significant change in time spent in play activity and in the reduction of Impulsive strategies. Initial session showed the child’s inability to sustain play activity, characterized by play segments of short duration, with the child going in and out of play activity frequently. The proportion of total time spent in play during the session was low, and the time spent in non-play was high. As treatment progressed, play activity segments increased in duration and the sessions flowed more smoothly, with fewer moves in and out of play and more time spent in play activity. At the end of treatment, the child showed no impulsive strategies, however, still showed significant Inhibited characteristics which were counterbalanced by adaptive strategies.

In terms of limitations, even though longitudinal studies of single cases are ideal to study the psychoanalytic process in depth, there is an issue with generalizing from a single case. Furthermore, it is important to underline the small number of time points pertaining to our three time series as a reasonable explanation for the lack of statistically significant odds ratios. An improved methodology would be based on a repeated single case design, preferably with more time points, involving relatively large sample of treatments for adequate comparison. Moreover, the use of outcome measures was limited in frequency and did not permit some statistical analyses that would be able to relate these measures to the CPTI. The intensive use of outcome measures, linked to the process measures, may bring further light to the psychodynamic play therapy process with children. Another limitation is that this study focused on the child’s use of the play space, however, we were not able to account for the therapists’ effects. Future studies can also apply other measures of process such as the Child Psychotherapy Q-Set (CPQ; Schneider et al., 2009) in order to understand core therapist factors and therapeutic interaction that aid in the emergence and development of play profiles. We also suggest that future studies could analyze play profiles of children with different ages, different pathologies, and different theoretical approaches which could yield different results pertaining to change processes.

Given the small sample size, and the limited ability to generalize findings, this study sought to put forth an initial empirical model that could be used to deepen our understanding of salient forces in psychodynamic play therapy in an innovative way. This was the first study of its kind to use CPTI with non-linear methods to systematically track play profiles in the therapeutic process, and analyzing their emergence and development. Approaching play processes as a multi-leveled phenomenon and categorizing the progression of different play profiles in psychodynamic psychotherapy can be used as a lens to study core processes of change. The methodology employed showed the productivity of treating psychodynamic play therapy as a complex system, taking advantage of a sophisticated outlook to study the process of play activity in treatment.

## Author Contributions

SH: Study conception and design, Acquisition of data, Interpretation of data, Drafting of manuscript, Finalizing Manuscript. AC: Study conception and design, Analysis and interpretation of data, Drafting of manuscript. FO: Analysis and interpretation of data, Drafting of manuscript. GS: Analysis and interpretation of data, Drafting of manuscript. SA: Analysis and interpretation of data, Drafting of manuscript. AG: Analysis and interpretation of data, Drafting of manuscript. GF: Study conception and design, Analysis and Interpretation of data, Drafting of manuscript, Finalizing Manuscript.

## Conflict of Interest Statement

The authors declare that the research was conducted in the absence of any commercial or financial relationships that could be construed as a potential conflict of interest.

## Footnotes

1 All potential identifying information on the patients, including names, was altered in order to protect her identity.
